# Subsite‐specific metastatic organotropism and risk in gastric cancer: A population‐based cohort study of the US SEER database and a Chinese single‐institutional registry

**DOI:** 10.1002/cam4.6583

**Published:** 2023-09-23

**Authors:** Ling Wang, Boxuan Liang, Yu Jiang, Genjie Huang, Aiwei Tang, Zhihong Liu, Yupeng Wang, Rui Zhou, Nanyan Yang, Jianhua Wu, Min Shi, Jianping Bin, Yulin Liao, Wangjun Liao

**Affiliations:** ^1^ Department of Oncology Nanfang Hospital, Southern Medical University Guangzhou China; ^2^ Guangdong Province Key Laboratory of Molecular Tumor Pathology Guangzhou China; ^3^ Department of Neurology Affiliated Dongguan Hospital, Southern Medical University Dongguan China; ^4^ Department of Cardiology Nanfang Hospital, Southern Medical University Guangzhou China

**Keywords:** gastric cancer, metastatic management, metastatic organotropism, metastatic risk, primary tumor location

## Abstract

**Background:**

Studies exploring whether metastatic organotropism and risk in gastric cancer (GC) differ by primary anatomical site are scarce.

**Methods:**

This study included 15,260 and 1623 patients diagnosed with GC from the Surveillance, Epidemiology, and End Results (SEER) registry database and the Nanfang Hospital in China, respectively. Patients were stratified according to primary site of GC, and the incidence of metastasis to different organs was used to determine the metastatic organotropism for each GC subsite. Finally, the metastatic organotropism and risk were compared among the different subsite groups.

**Results:**

Liver metastasis was the most common metastasis site in cardia GC, whereas other‐site metastases were more common in the body, antrum, overlapping lesions, and unspecified GCs. Liver and other‐site metastases were also frequently observed in the fundus, pylorus, lesser curvature, and greater curvature GCs. Patients with GC with definite primary tumor sites in the SEER and validation Nanfang hospital cohorts were compared by grouping as proximal and distal GCs for further analysis. In the SEER cohort, the top three metastatic sites of proximal GC were liver (21.4%), distant lymph node (LN) (14.6%), and other‐site (mainly peritoneum, 11.9%), whereas those of distal GC were other‐site (mainly peritoneum, 19.5%), liver (11.8%), and distant LN (9.5%). The incidence of metastasis to the liver, distant LN, lung, and brain was significantly higher in patients with proximal GC than in those with distal GC in both the SEER and Nanfang cohorts (*p* < 0.05). However, metastasis to other‐site/peritoneum was significantly lower in patients with proximal GC compared to those with distal GC in the Nanfang Hospital and SEER cohorts, respectively (*p* < 0.05).

**Conclusion:**

Liver and distant LN are the preferred metastatic sites for proximal GC, whereas peritoneal metastasis is more common in distal GC. Proximal GC has a higher risk of lymphatic and hematogenous metastases, and a lower risk of transcoelomic metastasis than distal GC. Our findings highlight the need to stratify GC by its primary subsite to aid in planning and decision‐making related to metastatic management in clinical practice.

## Graphical abstract

Metastatic organotropism and risk vary by primary anatomical subsite in gastric cancer (GC). Liver and distant lymph nodes are the preferred metastatic sites for proximal GC, whereas peritoneal metastasis is more common in distal GC. Proximal GC has a higher risk of lymphatic and hematogenous metastases and a lower risk of transcoelomic metastasis, compared to distal GC.

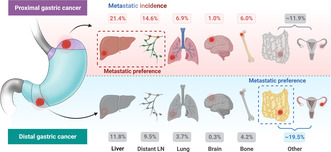

## 1 INTRODUCTION

Gastric cancer (GC) is a major contributor to the global cancer burden. In 2020, about 1,089,103 new GC cases were reported worldwide, making GC the fifth most common malignancy.^1^ Approximately one‐third of GC cases are diagnosed as Stage III or IV. Despite rapid advancements in treatment methods in recent years, the prognostic outcomes of patients with metastatic GC remain poor. Distant metastasis of cancer is associated with poor prognosis and is the leading cause of cancer‐related death,^2^ with almost 90% of patients dying by succumbing to metastasis.^3^ Therefore, an in‐depth understanding of distant metastatic patterns is beneficial for guiding workup and surveillance strategies in clinical practice.

With rapid advancements in genetic and molecular subtyping, the oncology community is becoming increasingly interested in the relationship between primary tumor location and metastasis.^4^, ^5^, ^6^ Primary tumor sites are highly correlated with metastatic organotropism. This subsite‐specific metastatic organotropism has been observed in colon cancer, where liver metastases are most common in patients with left colon cancer, and peritoneal and lung metastases are most common in patients with right colon and rectal cancers, respectively.^7^ Subsite‐specific metastatic organotropism has also been observed in Merkel cell carcinoma, in which the distant metastatic pattern differs according to its primary sites.^8^ However, little is currently known about whether the primary subsite of GC influences its distant metastasis outcomes and therefore affects its prognosis.

The trends of GC incidence are shifting divergently by anatomical subsite,^9^, ^10^ making it clinically beneficial to explore the potential relationship between the primary subsite of GC and its metastatic organotropism and risk. To do so, we analyzed the clinical data from the US National Cancer Institute's Surveillance, Epidemiology, and End Results (SEER) database and validated it using an external Chinese single‐institutional cohort. Our results emphasize the potential importance of stratifying GC by primary subsite for tumor metastatic management in clinical practice.

## 2 METHODS

### 2.1 Database and study population

The study data were obtained from two sources: the SEER Program and a Chinese single‐institutional registry. The SEER dataset, which represents the US population, was extracted from 17 geographically diverse populations between January 1, 2016, and December 31, 2019. Data from a single‐institutional registry of patients with GC who sought treatment at Nanfang Hospital in China between January 1, 2019 and December 31, 2021, were also included for the analyses, representing the Chinese population. The World Health Organization classification was used to define the following anatomical subsites: cardia, fundus, corpus, antrum, pylorus, lesser curvature, greater curvature, overlapping, and unspecified. The American Joint Committee on Cancer 8th edition staging criteria were used to define distant metastases as any clinically, pathologically, or radiologically confirmed GC found beyond the draining lymph nodes (LNs) of the primary lesion site. We excluded cases according to the following exclusion criteria: (a) unknown age, survival time, and metastatic status; (b) diagnosis by autopsy and death certificate; (c) multiple primary malignant tumors; and (d) pathological type confirmed to be neuroendocrine carcinoma (NEC), gastrointestinal stromal tumor (GIST), Kaposi sarcoma (KS), sarcoma, or lymphoma (Figure S1). The data from SEER were deidentified and publicly available under a data use agreement with the US National Cancer Institute. Therefore, this part of the study is exempt from a full board review by the institutional review boards of the participating research institutions. Ethical approval for the study of the Nanfang Hospital cohort was granted by the Medical Ethics Committee of the Nanfang Hospital.

### 2.2 Statistical analyses

Fisher's exact test was used to identify distant metastatic sites that differed between groups. Logistic regression models were employed to control for potential confounding variables, including age, sex, race, grade, chemotherapy, surgery, and radiation, and to estimate the probability of metastases. Statistical analyses were performed using SPSS Statistics version 25.0 (IBM Corp.). Categorical data were compared using Fisher's exact test, with statistical significance defined as a *p* < 0.05.

## 3 RESULTS

### 3.1 Characteristics of patients from the SEER cohort

A total of 15,260 patients with GC (male, 9718 [63.7%]; female, 5542 [36.3%]) from the SEER cohort were enrolled in this study. Gastric cardia cancer takes up the highest proportion (33.6%), followed by gastric antrum cancer (17.9%), and gastric body cancer (10.1%). Overlapping lesion cancers and GCs with unspecified primary sites accounted for 7.7% and 14.0% of GCs, respectively. Significant differences were observed in the distribution of sex, age, race, grade, and Lauren classification among patients with GC with different primary sites. Male patients accounted for a higher proportion (78.1%) of patients with gastric cardiac cancer. Patients diagnosed at ages 60–79 years, white race, and with intestinal type tumors made up the majority of patients in the SEER cohort. Table 1 shows the detailed baseline clinical characteristics of the patients.

**TABLE 1:** Characteristics of patients from the SEER cohorts.

Features	Total	Cardia, NOS	Fundus	Body	Gastric antrum	Pylorus	Lesser curvature	Greater curvature	Overlapping lesion	Stomach, NOS	*p*
Total	15,260	5130 (33.6%)	536 (3.5%)	1546 (10.1%)	2731 (17.9%)	436 (2.9%)	1065 (7.0%)	505 (3.3%)	1180 (7.7%)	2132 (14.0%)	
Sex											
Female	5542 (36.3%)	1124 (21.9%)	213 (39.7%)	718 (46.4%)	1212 (44.4%)	174 (39.9%)	424 (39.8%)	229 (45.3%)	509 (43.1%)	939 (44%)	<0.001
Male	9718 (63.7%)	4006 (78.1%)	323 (60.3%)	828 (53.6%)	1519 (55.6%)	262 (60.1%)	640 (60.2%)	276 (54.7%)	671 (56.9%)	1193 (56%)	
Age at diagnose (year)											
18–39	704 (4.6%)	169 (3.3%)	18 (3.4%)	99 (6.4%)	93 (3.4%)	26 (6%)	46 (4.3%)	28 (5.5%)	70 (5.9%)	155 (7.3%)	<0.001
40–59	3966 (26.0%)	1303 (25.4%)	147 (27.4%)	413 (26.7%)	612 (22.4%)	111 (25.5%)	267 (25.1%)	141 (27.9%)	330 (28%)	642 (30.1%)	
60–79	7901 (51.8%)	2990 (58.3%)	280 (52.2%)	734 (47.5%)	1377 (50.4%)	205 (47%)	539 (50.7%)	249 (49.3%)	569 (48.2%)	958 (44.9%)	
>80	2689 (17.6%)	668 (13.0%)	91 (17%)	300 (19.4%)	649 (23.8%)	94 (21.6%)	212 (19.9%)	87 (17.2%)	211 (17.9%)	377 (17.7%)	
Race											
White	10,629 (69.7%)	4408 (85.9%)	398 (74.3%)	1004 (64.9%)	1489 (54.5%)	240 (55.0%)	483 (56.6%)	314 (62.2%)	765 (64.8%)	1422 (66.7%)	<0.001
Asian or Pacific Islander	2540 (16.6%)	392 (7.6%)	58 (10.8%)	301 (19.5%)	723 (26.5%)	93 (21.3%)	589 (55.4%)	104 (20.6%)	233 (19.7%)	347 (16.3%)	
Black	1479 (12.4%)	270 (5.3%)	59 (11.0%)	201 (13.0%)	462 (16.9%)	85 (19.5%)	156 (14.7%)	69 (13.7%)	151 (12.8%)	293 (13.7%)	
American Indian/Alaska Native	1746 (11.4%)	30 (0.6%)	11 (2.1%)	17 (1.1%)	22 (0.8%)	12 (2.8%)	15 (1.4%)	8 (1.6%)	22 (1.9%)	35 (1.6%)	
Unknown	172 (1.1%)	30 (0.6%)	10 (1.9%)	23 (1.5%)	35 (1.3%)	6 (1.4%)	15 (1.4%)	10 (2.0%)	9 (0.8%)	35 (1.6%)	
Grade											
1	476 (3.1%)	199 (3.9%)	13 (2.4%)	42 (2.7%)	90 (3.3%)	16 (3.7%)	44 (4.1%)	18 (3.6%)	22 (1.9%)	32 (1.5%)	<0.001
2	3004 (19.7%)	1365 (26.6%)	113 (21.1%)	231 (14.9%)	513 (18.8%)	93 (21.3%)	210 (19.7%)	85 (16.8%)	172 (14.6%)	222 (10.4%)	
3	8394 (55.0%)	2501 (48.8%)	283 (52.8%)	1015 (65.7%)	1527 (55.9%)	226 (51.8%)	611 (57.4%)	284 (56.2%)	771 (65.3%)	1176 (55.2%)	
4	102 (0.7%)	20 (0.4%)	7 (1.3%)	16 (1%)	25 (0.9%)	3 (0.7%)	9 (0.8%)	4 (0.8%)	7 (0.6%)	11 (0.5%)	
Unknown	3284 (21.5%)	1045 (20.4%)	120 (22.4%)	242 (15.7%)	576 (21.1%)	98 (22.5%)	190 (17.9%)	114 (22.6%)	208 (17.6%)	691 (32.4%)	
Lauren classification											
Intestinal	10,681 (70%)	4262 (83.1%)	386 (72%)	936 (60.5%)	1780 (65.2%)	298 (68.3%)	733 (68.9%)	313 (62%)	642 (54.4%)	1331 (62.4%)	<0.001
Diffused	3725 (24.4%)	537 (10.5%)	119 (22.2%)	536 (34.7%)	797 (29.2%)	112 (25.7%)	270 (25.4%)	154 (30.5%)	483 (40.9%)	717 (33.6%)	
Unspecified	854 (5.6%)	331 (6.5%)	31 (5.8%)	74 (4.8%)	154 (5.6%)	26 (6%)	61 (5.7%)	38 (7.5%)	55 (4.7%)	84 (3.9%)	
Chemotherapy											
Yes	9135 (5.9%)	3529 (68.8%)	316 (59.0%)	905 (58.5%)	1399 (51.2%)	245 (56.2%)	615 (57.8%)	288 (57.0%)	752 (63.7%)	1086 (50.9%)	<0.001
No/unkown	5125 (40.1%)	1601 (31.2%)	220 (41.0%)	641 (41.5%)	1332 (48.8%)	191 (43.8%)	449 (42.2%)	217 (43.0%)	428 (36.3%)	1046 (49.1%)	
Surgery											
Yes	5981 (39.2%)	1836 (35.8%)	191 (35.6%)	649 (42%)	1345 (49.2%)	255 (58.5%)	545 (51.2%)	227 (45%)	456 (38.6%)	477 (22.4%)	<0.001
No/unkown	9279 (60.8%)	3294 (64.2%)	345 (64.4%)	897 (58%)	1386 (50.8%)	181 (41.5%)	519 (48.8%)	278 (55%)	724 (61.4%)	1655 (77.6%)	
Radiation											
Yes	3621 (23.7%)	2218 (43.2%)	84 (15.7%)	210 (13.6%)	384 (14.1%)	80 (18.3%)	161 (15.1%)	61 (12.1%)	186 (15.8%)	237 (11.1%)	<0.001
No/unkown	11,639 (76.3%)	2912 (56.8%)	452 (84.3%)	1336 (86.4%)	2347 (85.9%)	356 (81.7%)	903 (84.9%)	444 (87.9%)	994 (84.2%)	1895 (88.9%)	

### 3.2 Metastatic organotropism varies by primary anatomical subsite in gastric cancer

**FIGURE 1:**
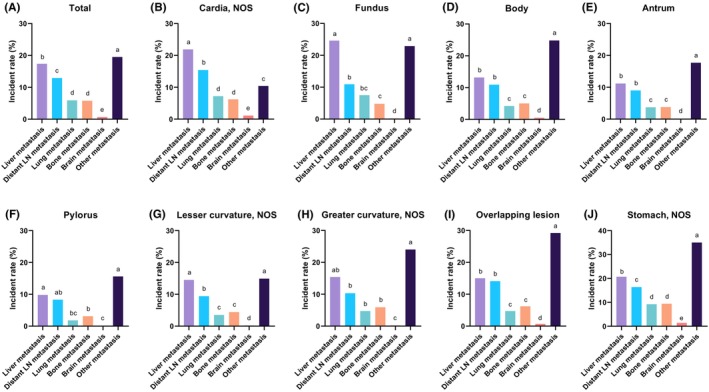
The metastatic incidences to different sites in gastric cancers with different primary anatomical subsites. The metastatic incidences of gastric cancer originated in (A) all, (B) cardia, (C) fundus, (D) body, (E) antrum, (F) pylorus, (G) lesser curvature, (H) greater curvature, (I) overlapping lesion, and (J) not otherwise specified location in the SEER cohort. The presence of the different lowercase letter above the bars denotes a significant difference between groups (*p* < 0.05).

Patients were stratified according to their primary tumor site, and the incidence of metastasis to different organs was compared and rank in each subtype of GC. Among the five definite distant metastatic locations (i.e., liver, distant LN, lung, bone, and brain) documented by the SEER database, liver (17.4%) was the leading metastatic site in GCs, followed by distant LN (12.9%), lung (5.9%), and bone (5.8%). Only a few metastases were present in the brains of patients with GC (0.7%). When all distant metastatic locations were considered, the metastatic incidence at other locations (19.5%) was higher than that of the definite metastatic locations mentioned above (Figure 1A). Metastatic organotropism varies by primary anatomical subsite in gastric cancer (GC). Although the other‐site metastasis included multiple metastatic sites in the calculation, it is worth noticing that the other‐site metastasis was the most prevalent metastasis in all subgroups of GC, except for cardia GC (Figure 1B–J). In particular, liver metastasis was the most prevalent in cardia GCs (21.26%) (Figure 1B). Other‐site metastasis was observed in the body (23.0%), antrum (16.8%), overlapping lesions (27.5%), and not otherwise specified (NOS) GCs (33.4%) (Figure 1D,E,I,J). Moreover, both the liver and other‐site metastasis topped the metastatic rates in the fundus (24.6% and 21.7%, respectively), pylorus (9.8% and 15.0%, respectively), lesser curvature (14.5% and 14.3%, respectively), and greater curvature GCs (15.4% and 23.5%, respectively) (Figure 1C, F, G, H).

### 3.3 Distant metastatic risk of GC differs by primary tumor sites

**FIGURE 2:**
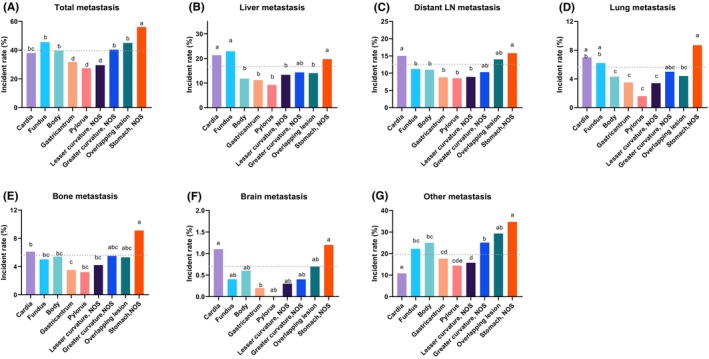
The metastatic incidences among gastric cancers with different primary anatomical subsites. The risk of (A) all, (B) liver, (C) distant lymph nodes, (D) lung, (E) bone, (F) brain, and (G) other‐site metastases among gastric cancers with different primary anatomical subsites in the SEER cohort. The dashed line indicates the average metastatic incidence in each organ. The presence of the different lowercase letter above the bars denotes a significant difference between groups (*p* < 0.05).

The risk of distant metastases was compared among GCs with different primary anatomical subsites. As we could not identify the specific locations of the overlapping lesion and NOS GCs, the results of these two types of GCs were shown, but were not within the scope of our current analysis. The overall metastatic incidences of GCs originating in the cardia, fundus, body, and greater curvature were significantly higher than those originating in the antrum, pylorus, and lesser curvature (*p* < 0.05, Figure 2A). We found that the cardia and fundus primary were associated with higher risks for liver and lung metastases compared with the body, antrum, pylorus, and lesser curvature primary (*p* < 0.05, Figure 2B,D). The cardia primary was associated with a higher risk for distant LN metastasis compared with the fundus, body, antrum, pylorus, and lesser curvature primary (*p* < 0.05, Figure 2C). For bone and brain metastases, the metastatic incidence of cardia GC was higher than that of antrum GC (*p* < 0.05, Figure 2E,F). Conversely, the cardia primary was associated with the lowest risk for other‐site metastasis among all subgroups (*p* < 0.05, Figure 2G). Taken together, we found the distant metastatic risks of GCs differs by their primary sites.

### 3.4 Characteristics of patients with definite primary tumor sites from the Nanfang Hospital and SEER cohorts

We enrolled 1623 patients with GC with definite primary tumor sites from Nanfang Hospital to verify whether the organotropism and risk of distant metastases of GC differs by primary tumor site. In light of the similar metastatic organotropism in GCs originating from adjacent sites, the patients in the Nanfang Hospital and SEER cohorts were further classified as proximal GC, which included cardia and fundus GCs, and distal GC, which included body, antrum, pylorus, lesser curvature, and greater curvature GCs. Thus, a total of 11,948 GC patients with definite primary tumor sites were included in the SEER cohort. Specifically, we found that the Nanfang Hospital cohort mainly consisted of distal GC (75.8%), whereas the proportions of proximal GCs were similar to those of distal GCs in the SEER cohort (47.4% vs. 52.6%). The sex ratio, age, Lauren classification, and grade compositions of patients with proximal and distal GCs were different in both cohorts (Table 2). Additionally, the race composition of patients with proximal and distal GCs was also different in the SEER cohort, whereas the patients enrolled from Nanfang Hospital were all Asian. The detailed baseline clinical characteristics of the two cohorts are shown in Table 2.

**TABLE 2:** Characteristics of patients with definite primary tumor sites from the Nanfang Hospital and SEER cohorts.

Features	SEER				Nanfang Hospital			
	Total	Proximal	Distal	*p*	Total	Proximal	Distal	*p*
Total	11,948	5666 (47.4%)	6282 (52.6%)		1623	392 (24.2%)	1231 (75.8%)	
Sex								
Female	4094 (34.3%)	1337 (23.6%)	2757 (43.9%)	<0.001	541 (33.3%)	78 (19.9%)	463 (37.6%)	<0.001
Male	7854 (65.7%)	4329 (76.4%)	3525 (56.1%)		1082 (66.7%)	314 (80.1%)	768 (62.4%)	
Age at diagnose (year)								
18–39	479 (4.0%)	187 (3.3%)	292 (4.6%)	<0.001	161 (9.9%)	15 (3.8%)	146 (11.9%)	<0.001
40–59	2994 (25.1%)	1450 (25.6%)	1544 (24.6%)		693 (42.7%)	129 (32.9%)	564 (45.8%)	
60–79	6374 (53.3%)	3270 (57.7%)	3104 (49.4%)		730 (45.0%)	236 (60.2%)	494 (40.1%)	
>80	2101 (17.6%)	759 (13.4)	1342 (21.4%)		39 (2.4%)	12 (3.1%)	27 (2.2%)	
Race								
White	8442 (70.7%)	4806 (84.8%)	3636 (57.9%)	<0.001	0	0	0	/
Asian or Pacific Islander	1960 (16.4%)	450 (7.9%)	1510 (24.0%)		1623 (100%)	392 (100%)	1231 (100%)	
Black	1302 (10.9%)	329 (5.5%)	973 (15.5%)		0	0	0	
American Indian/Alaska Native	115 (1.0%)	41 (0.7%)	74 (1.2%)		0	0	0	
Unknown	129 (1.1%)	40 (0.7%)	89 (1.4%)		0	0	0	
Grade								
1	422 (3.5%)	212 (3.7%)	210 (3.3%)	<0.001	98 (6.0%)	47 (12.0%)	51 (4.1%)	<0.001
2	2610 (21.8%)	1478 (26.1%)	1132 (18.0%)		284 (17.5%)	125 (31.9%)	159 (12.9%)	
3	6447 (54.0%)	2784 (49.1%)	3663 (58.3%)		1173 (72.3%)	198 (50.5%)	975 (79.2%)	
4	84 (0.7%)	27 (0.5%)	57 (0.9%)		3 (0.2%)	0 (0%)	3 (0.2%)	
Unknown	2385 (2.0%)	1165 (20.6%)	1220 (19.4%)		65 (4.0%)	22 (5.6%)	43 (3.5%)	
Lauren classification								
Intestinal	10,681 (70.0%)	4648 (82.0%)	4060 (64.6%)	<0.001	371 (22.9%)	152 (38.8%)	219 (17.8%)	<0.001
Diffused	3725 (24.4%)	656 (11.6%)	1869 (28.8%)		860 (53.0%)	101 (25.8%)	759 (61.7%)	
Unspecified	854 (5.6%)	363 (6.4%)	353 (5.6%)		392 (24.2%)	139 (35.5%)	253 (20.6%)	
Chemotherapy								
Yes	7297 (61.1%)	3845 (67.9%)	3452 (55.0%)	<0.001	999 (61.6%)	255 (65.1%)	744 (60.4%)	0.108
No/unkown	4651 (38.9%)	1821 (32.1%)	2830 (45.0%)		624 (38.4%)	137 (34.9)	487 (39.6%)	
Surgery								
Yes	5048 (42.2%)	2027 (35.8%)	3021 (48.1%)	<0.001	1082 (66.7%)	251 (64.0%)	831 (67.5%)	0.219
No/unkown	6900 (57.8%)	3639 (64.2%)	3261 (51.9%)		541 (33.3%)	141 (36.0%)	400 (32.5%)	
Radiation								
Yes	3198 (26.8%)	2302 (40.6%)	896 (14.3%)	<0.001	48 (3.0%)	25 (6.4%)	23 (1.9%)	<0.001
No/unkown	8750 (73.2%)	3364 (59.4%)	5386 (85.7%)		1575 (97.0%)	367 (93.6%)	1208 (98.1%)	

### 3.5 Proximal and distal GCs have different metastatic organotropism

**FIGURE 3:**
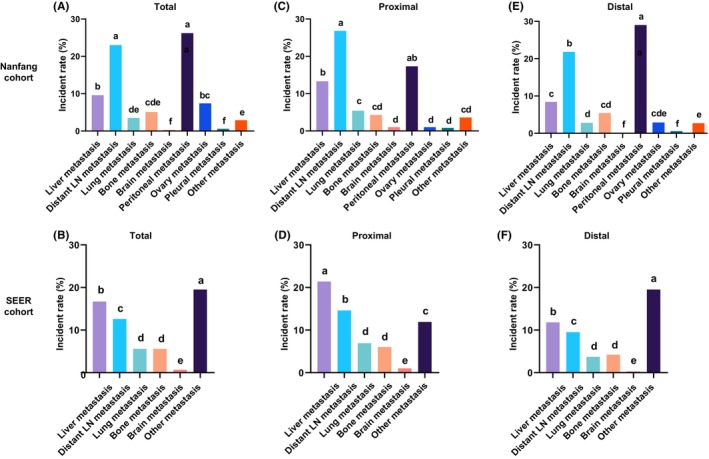
The metastatic incidences of proximal and distal gastric cancers in the Nanfang Hospital and SEER cohorts. The metastatic incidences of gastric cancer in (A) Nanfang Hospital and (B) SEER cohort; the metastatic incidences of proximal gastric cancer in (C) Nanfang Hospital and (D) SEER cohort; the metastatic incidences of distal gastric cancer in (E) Nanfang Hospital and (F) SEER cohort. The presence of the different lowercase letter above the bars denotes a significant difference between groups (*p* < 0.05).

Overall, peritoneal and distant LN metastases were the leading metastatic sites in GCs (26.2% and 23.0%, respectively) in the Nanfang Hospital cohorts, followed by the liver (9.6%), ovary (7.4%), bone (5.1%), and lung (3.5%). Only a few metastases were present in the pleural (0.6%) and brain (0.3%) of patients with GC (Figure 3A). Based on these results, we infer that the other metastatic sites in the SEER cohort mainly consist of peritoneal metastasis, combined with a small number of ovarian and pleural metastases. Based on this inference, we found consistency between the Nanfang Hospital and SEER cohorts that peritoneal, distant LN, and liver are the top three preferred metastatic sites of GC. However, it should be noticed that the liver metastatic incidence in the Nanfang Hospital cohort was significantly lower than that in the SEER cohort (Figure 3A,B). The metastatic incidences among different sites were compared in proximal and distal GCs in the Nanfang Hospital and SEER cohorts. Metastatic organotropism between proximal and distal GCs were different. Among all metastatic sites, the liver metastatic incidence was highest in proximal GC (21.4%, *p* < 0.05, Figure 3D), whereas the other metastatic incidence (mainly peritoneal metastasis) was highest in distal GC in the SEER cohort (19.5%, *p* < 0.05, Figure 3F). The metastatic incidence of distant LN in proximal GC was higher than that of peritoneal metastasis in the Nanfang Hospital (26.8% vs. 17.3%, *p* < 0.05, Figure 3C) and SEER (14.6% vs. 11.9%, *p* < 0.05, Figure 3D) cohorts. In contrast, the metastatic incidence of distant LN in distal GC was lower than that of peritoneal metastasis in the Nanfang Hospital (21.8% vs. 29.0%, *p* < 0.05, Figure 3E) and SEER (9.5% vs. 19.5%, *p* < 0.05, Figure 3F) cohorts. Taken together, our findings indicate that metastasis of proximal GC favors the liver and distant LN, whereas metastasis of distal GC favors the peritoneum.

### 3.6 Distant metastatic risk differs between proximal and distal GCs

**FIGURE 4:**
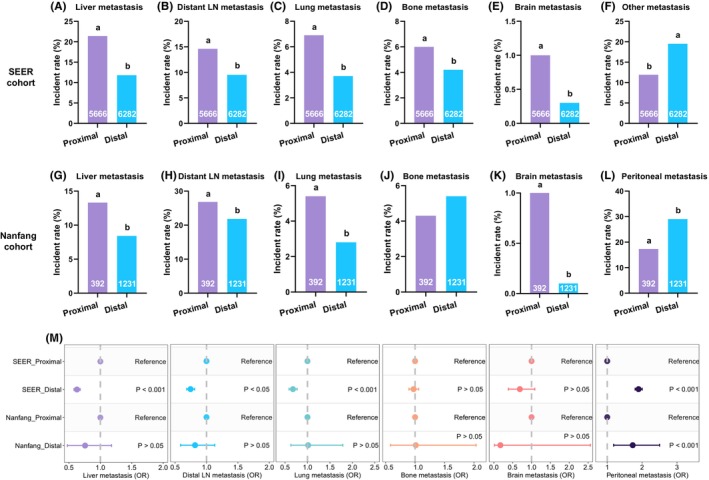
The metastatic incidences between proximal and distal gastric cancers. The risk of (A) liver, (B) distant lymph nodes, (C) lung, (D) bone, (E) brain, and (F) other‐site metastases between proximal and distal gastric cancers in the SEER cohort; the risk of (G) liver, (H) distant lymph nodes, (I) lung, (J) bone, (K) brain, and (L) peritoneal metastases between proximal and distal gastric cancers in the Nanfang Hospital cohort; (M) the odds ratios of metastatic incidences between proximal and distal gastric cancers in the logistic regression model adjusting for the potential confounding effects of age, sex, race, grade, chemotherapy, surgery, and radiation. The presence of the different lowercase letter above the bars denotes a significant difference between groups (*p* < 0.05).

The metastatic incidences at different sites were compared between proximal and distal GCs in the SEER and Nanfang Hospital cohorts. Consistent in the two cohorts, the metastatic incidences of the liver, distant LN, lung, and brain in patients with proximal GC were significantly higher than those in patients with distal GC, respectively (*p* < 0.05, Figure 4A–C,E,G–I,K). The metastatic incidences of the peritoneum and ovary in patients with proximal GC were significantly lower than those in patients with distal GC in the Nanfang Hospital cohort (*p* < 0.05, Figure 4L). Notably, the metastatic incidences of other‐site, which we infer are mainly consist of peritoneum, in patients with proximal GC were also significantly lower than those in patients with distal GC in the SEER cohort (*p* < 0.05, Figure 4F). Moreover, the metastatic incidence of the bone in patients with proximal GC was significantly higher than that in patients with distal GC in the SEER cohort (*p* < 0.05, Figure 4D), but not in the Nanfang Hospital cohort (*p >* 0.05, Figure 4J).

Furthermore, we used multivariable logistic regression to minimize the influence of potential confounding factors, including age, sex, race, grade, Lauren classification, chemotherapy, surgery, and radiation. We found that proximal GC showed a significantly higher metastatic risk in the liver (odds ratio [OR] = 1.51, 95% confidence interval [CI] = 1.34–1.70, *p* < 0.001), distant LN (OR = 1.33, 95% CI = 1.16–1.51, *p* < 0.001), and lung (OR = 1.62, 95% CI = 1.34–1.95, *p* < 0.001) in the SEER cohort compared with distant GC (Figure 4M; Tables S1, S3, and S5). However, these findings were not validated in the Nanfang Hospital cohort (liver: OR = 1.31, 95% CI = 0.86–1.99, *p* > 0.05; distant LN: OR = 1.19, 95% CI = 0.86–1.65, *p* > 0.05; lung: OR = 0.94, 95% CI = 0.50–1.78, *p* > 0.05; Figure 4M, Tables S2, S4, and S6). Moreover, we found that distal GC showed a significantly higher peritoneal metastatic risk compared with proximal GC in the SEER cohort (OR = 1.89, 95% CI = 1.67–2.14, *p* < 0.001, Figure 4M, Table S11). This finding was validated in the Nanfang Hospital cohort (OR = 1.79, 95% CI = 1.26–2.53, *p* < 0.01, Figure 4M; Table S12). In addition, there were no evidence of different metastatic risks in bone and brain in the SEER and Nanfang Hospital cohort (Figure 4M; Tables S7–S10) and in ovary and pleura in the Nanfang Hospital cohort between proximal and distal GC based on the logistic regression analysis (Figure S2; Tables S13 and S14).

## 4 DISCUSSION

Ever since the 133‐year‐old “seed‐and‐soil” hypothesis,^11^ metastatic organotropism has remained one of cancer's largest enigmas. Although cancer cells are able to escape from the primary tumor and travel randomly around the body, it remains unclear why their invasive fingerprints differ among cancer types.^12^, ^13^ Moreover, these organ‐specific metastases are widely present in tumors of the same type but originate in different subsites.^7^, ^8^ Here, we demonstrate that subsite‐specific metastatic organotropism also exists in GC and that the distant metastatic risk of GC differs by primary tumor site, based on data from the SEER and Nanfang Hospital cohorts. Collectively, we found that proximal GC tended to have liver and distant LN metastases whereas distal GC preferred to have peritoneal metastasis. Compared to distal GC, proximal GC has a higher risk of lymphatic and hematogenous metastases, including distant LN, liver, lung, bone, and brain metastases, and a lower risk of transcoelomic metastasis, which mainly consisted of peritoneal and ovary metastases. Our findings extend the knowledge of GC metastatic patterns, which may contribute to workup and surveillance strategies in clinical practice.

Metastatic potential and preference may be determined by the genetic properties of the “seed”.^14^ Microsatellite‐unstable GCs are generally tumors with an antral location, whose frequency of LN metastasis is less than those not located antrally,^15^ which supports our findings. Moreover, the frequency of chromosomally unstable tumors increases in proximal GC.^16^ Chromosomal instability has pro‐metastatic effects and is associated with metastasis to specific organs.^14^, ^17^ This may partly explain why proximal GC is more prone to organ metastasis compared with distal GC. In addition, the gene expression of GC at different subsites is remarkably different, and these genes are mainly involved in extracellular matrix remodeling, angiogenesis, and metastasis.^18^ This may also help shape subsite‐specific metastatic organotropism in GC. Except for the “seed,” the “soil,” namely, tumor microenvironment, may also contribute to the determination of metastatic organotropism.^19^, ^20^

*Helicobacter pylori* infection is one of the most distinctive features that distinguishes the distal stomach from the proximal stomach, it helps form an inflammatory environment in the distal stomach.^21^ Inflammation increases the adherence of GC cells to human peritoneal mesothelial cells, which may eventually lead to peritoneal metastasis.^22^ Moreover, *H*. *pylori* affects multiple key steps in this cascade, resulting in peritoneal metastases. Specifically, *H. pylori* activates human neutrophils and promotes cell detachment.^23^ It facilitates the overexpression of the matrix metalloproteinase family, which is a major contributor to the process of stromal invasion.^24^, ^25^ Overall, *H. pylori* infection can be one of the reasons for facilitating metastasis of distal GC to the peritoneum.

The current study has several limitations. First, the anatomical subsites of GC were difficult to determine, which may have led to underreporting and misclassification. Although these challenges may persist, it would be valuable to determine potential solutions to identify the topography of GC cases, as metastatic organotropism may be affected by the primary subsites. Second, the highest metastasis category in the SEER cohort had ambiguous metastatic sites, which were categorized as other metastases. We inferred that the vast majority of this category were peritoneal metastasis, based on the data from our validation cohort and the fact that the peritoneum is one of the most common sites of metastasis in patients with GC.^26^ Although the specific value does not reflect peritoneal metastasis exactly in the SEER cohort, we believe this does not affect our main conclusion. Third, many confounding factors may influence our conclusions, such as the late occurrence of brain and bone metastasis in the disease course. The lower rate of brain and bone metastasis in distal GC may be attributed to its higher proportion of diffuse‐type GC, which is associated with poorer prognosis, thus affecting its rate of brain metastasis. While we used multivariable logistic regression to minimize the influence of potential confounding factors, some confounding factors, such as histological type, cell differentiation, depth of invasion, and tumor size, were not considered due to unavailability. Fourth, it is worth noting that time is another important factor that can influence tumor metastasis. However, due to the nature of this cross‐sectional study, we were unable to gather data on the specific timing of tumor metastasis events in each patient, thereby limiting our ability to evaluate the impact of time on tumor metastasis. Future studies should be conducted to specifically address this aspect. Finally, we used an external Chinese single‐institutional cohort to validate the findings from the SEER cohort. While we obtained consistent results indicating that distant metastatic risk differs between proximal and distal GCs, the results were not entirely consistent regarding metastatic organotropism. This could be due to the racial composition and relatively small sample size in the Nanfang Hospital cohort. Further studies with larger cohorts and multiple racial compositions are required.

## 5 CONCLUSION

**FIGURE 5:**
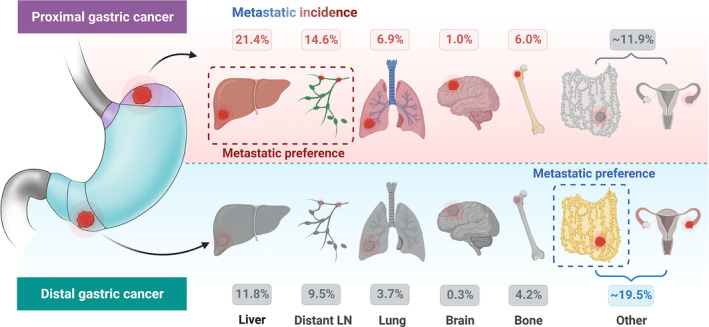
Schematic diagram of the present study. Metastatic organotropism and risk vary according to the anatomical subsite of GC. Liver and distant LNs are the preferred metastatic sites for proximal GC, whereas peritoneal metastasis is more common in distal GC. Proximal GC has a higher risk of lymphatic and hematogenous metastases, including distant LNs, liver, lung, bone, and brain metastases, and a lower risk of transcoelomic metastasis, which mainly consists of peritoneal and ovarian metastases, compared to distal GC.

Metastatic organotropism and risk vary according to the anatomical subsite of GC. Liver and distant LNs are the preferred metastatic sites for proximal GC, whereas peritoneal metastasis is more common in distal GC. Proximal GC has a higher risk of lymphatic and hematogenous metastases, including distant LNs, liver, lung, bone, and brain metastases, and a lower risk of transcoelomic metastasis, which mainly consists of peritoneal and ovarian metastases, compared to distal GC (Figure 5). Our findings suggest that the primary location of GC is closely associated with metastatic organotropism and risk, highlighting the need to stratify GC by its primary subsite to aid in planning and decision‐making related to metastatic management in clinical practice.

## AUTHOR CONTRIBUTIONS

**Ling Wang:** Conceptualization (equal); data curation (equal); formal analysis (equal); methodology (equal); project administration (equal); software (equal); visualization (equal); writing – original draft (equal). **Boxuan Liang:** Conceptualization (equal); data curation (equal); formal analysis (equal); investigation (equal); methodology (equal); project administration (equal); software (equal); validation (equal); visualization (equal); writing – original draft (equal). **Yu Jiang:** Investigation (equal); validation (equal). **Gen‐Jie Huang:** Methodology (equal); project administration (equal); visualization (equal). **Aiwei Tang:** Data curation (equal); software (equal). **Zhihong Liu:** Methodology (equal); validation (equal). **Yupeng Wang:** Formal analysis (equal); visualization (equal). **Rui Zhou:** Data curation (equal); investigation (equal). **Nanyan Yang:** Formal analysis (equal); visualization (equal). **Jianhua Wu:** Funding acquisition (equal); software (equal). **Min Shi:** Resources (equal); supervision (equal); writing – review and editing (equal). **Jianping Bin:** Resources (equal). **Yulin Liao:** Resources (equal). **Wangjun Liao:** Conceptualization (equal); funding acquisition (equal); resources (equal); supervision (equal); writing – review and editing (equal).

## FUNDING INFORMATION

This work was supported by the National Natural Science Foundation of China (No. 82073303, Wangjun Liao, No. 82002555, Jianhua Wu). The funders had no role in the design and conduct of the study; collection, management, analysis, and interpretation of the data; preparation, review, or approval of the manuscript; and decision to submit the manuscript for publication.

## CONFLICT OF INTEREST STATEMENT

The authors declare that they have no known competing financial interests or personal relationships that could have appeared to influence the work reported in this paper.

## Supporting information


Table S1.
Table S2.
Table S3.
Table S4.
Table S5.
Table S6.
Table S7.
Table S8.
Table S9.
Table S10.
Table S11.
Table S12.
Table S13.
Table S14.
Figure S1.
Figure S2.

## Data Availability

The authors commit to making the relevant anonymized patient level data available on reasonable request.
