# Supplementary material for: Subsite‐specific metastatic organotropism and risk in gastric cancer: A population‐based cohort study of the US SEER database and a Chinese single‐institutional registry
Source: Cancer Med. 2023 Sep 23;12(19):19595–606. doi: 10.1002/cam4.6583 (PMC10587925; doi:10.1002/cam4.6583)
Supplement: Supplementary file 1 — Table S1. Table S2. Table S3. Table S4. Table S5. Table S6. Table S7. Table S8. Table S9. Table S10. Table S11. Table S12. Table S13. Table S14. Figure S1. Figure S2. [file CAM4-12-19595-s001.docx]

**Subsite-specific metastatic organotropism and risk in gastric cancer: A population-based cohort study of the US SEER Database and a Chinese single-institutional registry**

Ling Wang^12^*, Boxuan Liang^3*^, Yu Jiang^1^, Genjie Huang^12^, Aiwei Tang^1^, Zhihong Liu^12^, Yupeng Wang^1^, Rui Zhou^12^, Nanyan Yang^1^, Jianhua Wu^12^, Min Shi^12^, Jianping Bin^4^, Yulin Liao^4^, Wangjun Liao^12#^

^1^Department of Oncology, Nanfang Hospital, Southern Medical University, Guangzhou, China.

^2^Guangdong Province Key Laboratory of Molecular Tumor Pathology, Guangzhou, China.

^3^Department of Neurology, Affiliated Dongguan Hospital, Southern Medical University, Dongguan, China.

^4^Department of Cardiology, Nanfang Hospital, Southern Medical University, Guangzhou, China.

* Ling Wang and Boxuan Liang contributed equally to this work as co-first authors.

^#^**Corresponding authors:**

Wangjun Liao. Department of Oncology, Nanfang Hospital, Southern Medical University, 1023-1063 Shatai Nan Road, Guangzhou 510515, China. Email address: [nfyyliaowj@163.com](mailto:nfyyliaowj@163.com).

**Supplementary material**

Table S1. Logistic analysis of liver metastasis in SEER cohort 3

Table S2. Logistic analysis of liver metastasis in Nanfang Hospital cohort 4

Table S3. Logistic analysis of distal LN metastasis in SEER cohort 5

Table S4. Logistic analysis of distal LN metastasis in Nanfang Hospital cohort 6

Table S5. Logistic analysis of lung metastasis in SEER cohort 7

Table S6. Logistic analysis of lung metastasis in Nanfang Hospital cohort 8

Table S7. Logistic analysis of bone metastasis in SEER cohort 9

Table S8. Logistic analysis of bone metastasis in Nanfang Hospital cohort 10

Table S9. Logistic analysis of brain metastasis in SEER cohort 11

Table S10. Logistic analysis of brain metastasis in Nanfang Hospital cohort 12

Table S11. Logistic analysis of other-site metastasis in SEER cohort 13

Table S12. Logistic analysis of peritoneal metastasis in Nanfang Hospital cohort 14

Table S13. Logistic analysis of ovary metastasis in Nanfang Hospital cohort 15

Table S14. Logistic analysis of pleura metastasis in Nanfang Hospital cohort 16

Figure S1. Flowchart of the patient selection process in SEER cohort of this study 17

Figure S2. The metastatic incidences of ovary and pleural between proximal and distal gastric cancers in the Nanfang Hospital cohort 17

| **Table S1. Logistic analysis of liver metastasis in SEER cohort** | | | | | |
| --- | --- | --- | --- | --- | --- |
| **Variable** | **Subgroup** | **OR** | **Lower** | **Upper** | ***P*** |
| Sex | Female | 1.000 | 1.000 | 1.000 |  |
|  | Male | .789 | .698 | .892 | .000 |
| Age | 18 – 39 | 1.000 | 1.000 | 1.000 |  |
|  | 40 – 59 | 1.956 | 1.428 | 2.680 | .000 |
|  | 60 – 79 | 1.903 | 1.585 | 2.284 | .000 |
|  | > 80 | 1.798 | 1.533 | 2.108 | .000 |
| Race | American | 1.000 | 1.000 | 1.000 |  |
|  | Asian or | 1.122 | .649 | 1.938 | .680 |
|  | Black | .872 | .736 | 1.033 | .113 |
|  | Unknown | 1.093 | .918 | 1.302 | .318 |
|  | White | .634 | .349 | 1.153 | .135 |
| Primary_site | Proximal | 1.000 | 1.000 | 1.000 |  |
|  | Distal | .664 | .589 | .749 | .0000 |
| Grade | 1 | 1.000 | 1.000 | 1.000 |  |
|  | 2 | .563 | .374 | .846 | .006 |
|  | 3 | 1.029 | .874 | 1.211 | .733 |
|  | 4 | .839 | .730 | .965 | .014 |
|  | Unknown | 2.271 | 1.140 | 4.524 | .020 |
| Lauren classification | Intestinal | 1.000 | 1.000 | 1.000 |  |
|  | Diffused | 1.481 | 1.158 | 1.893 | .002 |
|  | Unspecified | .302 | .223 | .410 | .000 |
| Chemotherapy | Yes | 1.000 | 1.000 | 1.000 |  |
|  | No/Unkown | .707 | .627 | .798 | .0000 |
| Surgery | Yes | 1.000 | 1.000 | 1.000 |  |
|  | No/Unkown | 24.297 | 19.245 | 30.677 | .0000 |
| Radiation | Yes | 1.000 | 1.000 | 1.000 |  |
|  | No/Unkown | 2.478 | 2.148 | 2.859 | .0000 |

| **Table S2. Logistic analysis of liver metastasis in Nanfang Hospital cohort** | | | | | |
| --- | --- | --- | --- | --- | --- |
| **Variable** | **Subgroup** | **OR** | **Lower** | **Upper** | ***P*** |
| Sex | Female | 1.000 | 1.000 | 1.000 |  |
|  | Male | 1.805 | 1.169 | 2.786 | .008 |
| Age | 18 – 39 | 1.000 | 1.000 | 1.000 |  |
|  | 40 – 59 | .979 | .292 | 3.290 | .973 |
|  | 60 – 79 | .771 | .260 | 2.288 | .639 |
|  | > 80 | 1.151 | .398 | 3.327 | .795 |
| Primary_site | Proximal | 1.000 | 1.000 | 1.000 |  |
|  | Distal | .766 | .503 | 1.165 | .213 |
| Grade | 1 | 1.000 | 1.000 | 1.000 |  |
|  | 2 | .326 | .115 | .925 | .035 |
|  | 3 | .522 | .261 | 1.043 | .066 |
|  | 4 | .280 | .153 | .513 | .000 |
|  | Unknown | .0000 | .0000 |  | 0.9990 |
| Lauren classification | Intestinal | 1.000 | 1.000 | 1.000 |  |
|  | Diffused | .744 | .439 | 1.262 | .273 |
|  | Unspecified | 1.247 | .803 | 1.936 | .326 |
| Chemotherapy | Yes | 1.0000 | 1.0000 | 1.0000 |  |
|  | No/Unkown | .388 | .254 | .591 | .000 |
| Surgery | Yes | 1.0000 | 1.0000 | 1.0000 |  |
|  | No/Unkown | 4.260 | 2.907 | 6.242 | .000 |
| Radiation | Yes | 1.0000 | 1.0000 | 1.0000 |  |
|  | No/Unkown | .676 | .299 | 1.529 | .347 |

| **Table S3. Logistic analysis of distal LN metastasis in SEER cohort** | | | | | |
| --- | --- | --- | --- | --- | --- |
| **Variable** | **Subgroup** | **OR** | **Lower** | **Upper** | ***P*** |
| Sex | Female | 1.000 | 1.000 | 1.000 |  |
|  | Male | .924 | .810 | 1.053 | .234 |
| Age | 18 – 39 | 1.000 | 1.000 | 1.000 |  |
|  | 40 – 59 | 2.670 | 1.958 | 3.641 | .000 |
|  | 60 – 79 | 1.954 | 1.591 | 2.399 | .000 |
|  | > 80 | 1.748 | 1.453 | 2.103 | .000 |
| Race | American | 1.000 | 1.000 | 1.000 |  |
|  | Asian or | 1.363 | .781 | 2.380 | .276 |
|  | Black | 1.239 | 1.043 | 1.471 | .015 |
|  | Unknown | .989 | .812 | 1.206 | .915 |
|  | White | .435 | .198 | .954 | .038 |
| Primary_site | Proximal | 1.000 | 1.000 | 1.000 |  |
|  | Distal | .754 | .661 | .859 | .0000 |
| Grade | 1 | 1.000 | 1.000 | 1.000 |  |
|  | 2 | .755 | .467 | 1.221 | .252 |
|  | 3 | .947 | .780 | 1.150 | .581 |
|  | 4 | 1.264 | 1.083 | 1.476 | .003 |
|  | Unknown | 1.653 | .787 | 3.474 | .185 |
| Lauren classification | Intestinal | 1.000 | 1.000 | 1.000 |  |
|  | Diffused | 1.082 | .830 | 1.410 | .561 |
|  | Unspecified | .700 | .520 | .942 | .019 |
| Chemotherapy | Yes | 1.000 | 1.000 | 1.000 |  |
|  | No/Unkown | .636 | .556 | .728 | .0000 |
| Surgery | Yes | 1.000 | 1.000 | 1.000 |  |
|  | No/Unkown | 21.232 | 16.241 | 27.757 | .0000 |
| Radiation | Yes | 1.000 | 1.000 | 1.000 |  |
|  | No/Unkown | 1.621 | 1.394 | 1.885 | .0000 |

| **Table S4. Logistic analysis of distal LN metastasis in Nanfang Hospital cohort** | | | | | |
| --- | --- | --- | --- | --- | --- |
| **Variable** | **Subgroup** | **OR** | **Lower** | **Upper** | ***P*** |
| Sex | Female | 1.000 | 1.000 | 1.000 |  |
|  | Male | 1.033 | .774 | 1.379 | .825 |
| Age | 18 – 39 | 1.000 | 1.000 | 1.000 |  |
|  | 40 – 59 | .910 | .359 | 2.307 | .842 |
|  | 60 – 79 | 1.057 | .450 | 2.480 | .899 |
|  | > 80 | 1.399 | .604 | 3.240 | .434 |
| Primary_site | Proximal | 1.000 | 1.000 | 1.000 |  |
|  | Distal | .838 | .605 | 1.161 | .289 |
| Grade | 1 | 1.000 | 1.000 | 1.000 |  |
|  | 2 | .471 | .189 | 1.174 | .106 |
|  | 3 | .809 | .423 | 1.549 | .523 |
|  | 4 | .988 | .561 | 1.738 | .966 |
|  | Unknown | .0000 | .0000 |  | 0.9990 |
| Lauren classification | Intestinal | 1.000 | 1.000 | 1.000 |  |
|  | Diffused | .866 | .588 | 1.277 | .469 |
|  | Unspecified | .947 | .687 | 1.306 | .741 |
| Chemotherapy | Yes | 1.0000 | 1.0000 | 1.0000 |  |
|  | No/Unkown | .396 | .294 | .534 | .000 |
| Surgery | Yes | 1.0000 | 1.0000 | 1.0000 |  |
|  | No/Unkown | 7.206 | 5.453 | 9.522 | .000 |
| Radiation | Yes | 1.0000 | 1.0000 | 1.0000 |  |
|  | No/Unkown | .308 | .157 | .602 | .347 |

| **Table S5. Logistic analysis of lung metastasis in SEER cohort** | | | | | |
| --- | --- | --- | --- | --- | --- |
| **Variable** | **Subgroup** | **OR** | **Lower** | **Upper** | ***P*** |
| Sex | Female | 1.000 | 1.000 | 1.000 |  |
|  | Male | .976 | .812 | 1.174 | . .800 |
| Age | 18 – 39 | 1.000 | 1.000 | 1.000 |  |
|  | 40 – 59 | 1.397 | .883 | 2.209 | .153 |
|  | 60 – 79 | 1.368 | 1.044 | 1.793 | .023 |
|  | > 80 | 1.212 | .956 | 1.537 | .112 |
| Race | American | 1.000 | 1.000 | 1.000 |  |
|  | Asian or | 1.453 | .689 | 3.065 | .327 |
|  | Black | 1.208 | .949 | 1.539 | .125 |
|  | Unknown | .932 | .700 | 1.241 | .630 |
|  | White | .443 | .139 | 1.415 | .169 |
| Primary_site | Proximal | 1.000 | 1.000 | 1.000 |  |
|  | Distal | .618 | .512 | .745 | .0000 |
| Grade | 1 | 1.000 | 1.000 | 1.000 |  |
|  | 2 | .744 | .393 | 1.409 | .364 |
|  | 3 | 1.153 | .902 | 1.474 | .255 |
|  | 4 | .829 | .671 | 1.025 | .083 |
|  | Unknown | 2.283 | .984 | 5.295 | .054 |
| Lauren classification | Intestinal | 1.000 | 1.000 | 1.000 |  |
|  | Diffused | 1.096 | .753 | 1.596 | .633 |
|  | Unspecified | .881 | .576 | 1.347 | .558 |
| Chemotherapy | Yes | 1.000 | 1.000 | 1.000 |  |
|  | No/Unkown | .950 | .790 | 1.142 | .585 |
| Surgery | Yes | 1.000 | 1.000 | 1.000 |  |
|  | No/Unkown | 17.109 | 11.577 | 25.284 | .0000 |
| Radiation | Yes | 1.000 | 1.000 | 1.000 |  |
|  | No/Unkown | 1.373 | 1.114 | 1.692 | .003 |

| **Table S6. Logistic analysis of lung metastasis in Nanfang Hospital cohort** | | | | | |
| --- | --- | --- | --- | --- | --- |
| **Variable** | **Subgroup** | **OR** | **Lower** | **Upper** | ***P*** |
| Sex | Female | 1.000 | 1.000 | 1.000 |  |
|  | Male | 1.362 | .689 | 2.695 | .374 |
| Age | 18 – 39 | 1.000 | 1.000 | 1.000 |  |
|  | 40 – 59 | .163 | .024 | 1.118 | .065 |
|  | 60 – 79 | .286 | .072 | 1.130 | .074 |
|  | > 80 | .498 | .135 | 1.838 | .295 |
| Primary_site | Proximal | 1.000 | 1.000 | 1.000 |  |
|  | Distal | 1.059 | .561 | 2.000 | .859 |
| Grade | 1 | 1.000 | 1.000 | 1.000 |  |
|  | 2 | .955 | .259 | 3.514 | .944 |
|  | 3 | .730 | .260 | 2.053 | .551 |
|  | 4 | .382 | .152 | .964 | .042 |
|  | Unknown | .0000 | .0000 |  | 0.9990 |
| Lauren classification | Intestinal | 1.000 | 1.000 | 1.000 |  |
|  | Diffused | .742 | .360 | 1.530 | .419 |
|  | Unspecified | .505 | .251 | 1.015 | .055 |
| Chemotherapy | Yes | 1.0000 | 1.0000 | 1.0000 |  |
|  | No/Unkown | .433 | .222 | .846 | .014 |
| Surgery | Yes | 1.0000 | 1.0000 | 1.0000 |  |
|  | No/Unkown | 2.787 | 1.531 | 5.072 | .001 |
| Radiation | Yes | 1.0000 | 1.0000 | 1.0000 |  |
|  | No/Unkown | .322 | .121 | .859 | .024 |

| **Table S7. Logistic analysis of bone metastasis in SEER cohort** | | | | | |
| --- | --- | --- | --- | --- | --- |
| **Variable** | **Subgroup** | **OR** | **Lower** | **Upper** | ***P*** |
| Sex | Female | 1.000 | 1.000 | 1.000 |  |
|  | Male | .948 | .786 | 1.144 | .580 |
| Age | 18 – 39 | 1.000 | 1.000 | 1.000 |  |
|  | 40 – 59 | 4.556 | 2.923 | 7.101 | .000 |
|  | 60 – 79 | 3.802 | 2.760 | 5.238 | .000 |
|  | > 80 | 2.548 | 1.882 | 3.450 | .000 |
| Race | American | 1.000 | 1.000 | 1.000 |  |
|  | Asian or | 1.298 | .611 | 2.758 | .498 |
|  | Black | 1.007 | .779 | 1.302 | .956 |
|  | Unknown | .955 | .718 | 1.271 | .754 |
|  | White | .577 | .209 | 1.597 | .290 |
| Primary_site | Proximal | 1.000 | 1.000 | 1.000 |  |
|  | Distal | .948 | .784 | 1.147 | .584 |
| Grade | 1 | 1.000 | 1.000 | 1.000 |  |
|  | 2 | .665 | .328 | 1.347 | .258 |
|  | 3 | .669 | .496 | .903 | .009 |
|  | 4 | 1.130 | .912 | 1.399 | .264 |
|  | Unknown | 1.493 | .566 | 3.941 | .418 |
| Lauren classification | Intestinal | 1.000 | 1.000 | 1.000 |  |
|  | Diffused | .933 | .641 | 1.359 | .719 |
|  | Unspecified | 1.172 | .781 | 1.757 | .444 |
| Chemotherapy | Yes | 1.000 | 1.000 | 1.000 |  |
|  | No/Unkown | 1.220 | 1.011 | 1.474 | .038 |
| Surgery | Yes | 1.000 | 1.000 | 1.000 |  |
|  | No/Unkown | 23.871 | 15.520 | 36.713 | .000 |
| Radiation | Yes | 1.000 | 1.000 | 1.000 |  |
|  | No/Unkown | .514 | .425 | .623 | .000 |

| **Table S8. Logistic analysis of bone metastasis in Nanfang Hospital cohort** | | | | | |
| --- | --- | --- | --- | --- | --- |
| **Variable** | **Subgroup** | **OR** | **Lower** | **Upper** | ***P*** |
| Sex | Female | 1.000 | 1.000 | 1.000 |  |
|  | Male | .893 | .543 | 1.470 | .657 |
| Age | 18 – 39 | 1.000 | 1.000 | 1.000 |  |
|  | 40 – 59 | 2.922 | .348 | 24.554 | .324 |
|  | 60 – 79 | 2.600 | .331 | 20.429 | .364 |
|  | > 80 | 2.279 | .292 | 17.799 | .432 |
| Primary_site | Proximal | 1.000 | 1.000 | 1.000 |  |
|  | Distal | 1.094 | .586 | 2.043 | .778 |
| Grade | 1 | 1.000 | 1.000 | 1.000 |  |
|  | 2 | .234 | .027 | 2.066 | .191 |
|  | 3 | .389 | .119 | 1.277 | .120 |
|  | 4 | .818 | .341 | 1.963 | .653 |
|  | Unknown | .0000 | .0000 |  | 0.999 |
| Lauren classification | Intestinal | 1.000 | 1.000 | 1.000 |  |
|  | Diffused | .742 | .360 | 1.530 | .998 |
|  | Unspecified | .999 | .474 | 2.104 | .297 |
| Chemotherapy | Yes | 1.352 | .767 | 2.383 |  |
|  | No/Unkown | .520 | .301 | .901 | .020 |
| Surgery | Yes | 1.0000 | 1.0000 | 1.0000 |  |
|  | No/Unkown | 5.232 | 3.125 | 8.759 | .000 |
| Radiation | Yes | 1.0000 | 1.0000 | 1.0000 |  |
|  | No/Unkown | .244 | .104 | .571 | .001 |

| **Table S9. Logistic analysis of brain metastasis in SEER cohort** | | | | | |
| --- | --- | --- | --- | --- | --- |
| **Variable** | **Subgroup** | **OR** | **Lower** | **Upper** | ***P*** |
| Sex | Female | 1.000 | 1.000 | 1.000 |  |
|  | Male | .894 | .517 | 1.547 | .690 |
| Age | 18 – 39 | 1.000 | 1.000 | 1.000 |  |
|  | 40 – 59 | 11.890 | 3.867 | 36.556 | .000 |
|  | 60 – 79 | 3.717 | 1.439 | 9.598 | .007 |
|  | > 80 | 3.438 | 1.423 | 8.305 | .006 |
| Race | American | 1.000 | 1.000 | 1.000 |  |
|  | Asian or | 4.299 | 1.242 | 14.879 | .021 |
|  | Black | .628 | .247 | 1.596 | .328 |
|  | Unknown | .857 | .360 | 2.036 | .726 |
|  | White | 1.963 | .262 | 14.698 | .511 |
| Primary_site | Proximal | 1.000 | 1.000 | 1.000 |  |
|  | Distal | .615 | .344 | 1.100 | .101 |
| Grade | 1 | 1.000 | 1.000 | 1.000 |  |
|  | 2 | .625 | .139 | 2.807 | .540 |
|  | 3 | .848 | .422 | 1.702 | .642 |
|  | 4 | .814 | .456 | 1.452 | .485 |
|  | Unknown | 2.138 | .262 | 17.432 | .478 |
| Lauren classification | Intestinal | 1.000 | 1.000 | 1.000 |  |
|  | Diffused | 4.956 | .678 | 36.251 | .115 |
|  | Unspecified | 5.831 | .747 | 45.508 | .093 |
| Chemotherapy | Yes | 1.000 | 1.000 | 1.000 |  |
|  | No/Unkown | 2.513 | 1.497 | 4.218 | .000 |
| Surgery | Yes | 1.000 | 1.000 | 1.000 |  |
|  | No/Unkown | 6.969 | 3.299 | 14.726 | .000 |
| Radiation | Yes | 1.000 | 1.000 | 1.000 |  |
|  | No/Unkown | .103 | .060 | .179 | .000 |

| **Table S10. Logistic analysis of brain metastasis in Nanfang Hospital cohort** | | | | | |
| --- | --- | --- | --- | --- | --- |
| **Variable** | **Subgroup** | **OR** | **Lower** | **Upper** | ***P*** |
| Sex | Female | 1.000 | 1.000 | 1.000 |  |
|  | Male | .893 | .543 | 1.470 | .657 |
| Age | 18 – 39 | 1.000 | 1.000 | 1.000 |  |
|  | 40 – 59 | 2.922 | .348 | 24.554 | .324 |
|  | 60 – 79 | 2.600 | .331 | 20.429 | .364 |
|  | > 80 | 2.279 | .292 | 17.799 | .432 |
| Primary_site | Proximal | 1.000 | 1.000 | 1.000 |  |
|  | Distal | .238 | .021 | 2.653 | .243 |
| Grade | 1 | 1.000 | 1.000 | 1.000 |  |
|  | 2 | .000 | .000 | . | .996 |
|  | 3 | .107 | .006 | 1.750 | .117 |
|  | 4 | .061 | .004 | .938 | .045 |
|  | Unknown | .0000 | .0000 |  | 0.999 |
| Lauren classification | Intestinal | 1.000 | 1.000 | 1.000 |  |
|  | Diffused | 4.282 | .329 | 55.772 | .267 |
|  | Unspecified | 1.001 | .044 | 22.935 | .999 |
| Chemotherapy | Yes | 1.352 | .767 | 2.383 |  |
|  | No/Unkown | 1.971 | .197 | 19.707 | .563 |
| Surgery | Yes | 1.0000 | 1.0000 | 1.0000 |  |
|  | No/Unkown | 1.874 | .174 | 20.161 | .604 |
| Radiation | Yes | 1.0000 | 1.0000 | 1.0000 |  |
|  | No/Unkown | .037 | .003 | .488 | .012 |

| **Table S11. Logistic analysis of other site metastasis in SEER cohort** | | | | | |
| --- | --- | --- | --- | --- | --- |
| **Variable** | **Subgroup** | **OR** | **Lower** | **Upper** | ***P*** |
| Sex | Female | 1.000 | 1.000 | 1.000 |  |
|  | Male | 1.322 | 1.177 | 1.484 | .000 |
| Age | 18 – 39 | 1.000 | 1.000 | 1.000 |  |
|  | 40 – 59 | 5.351 | 4.048 | 7.075 | .000 |
|  | 60 – 79 | 3.273 | 2.697 | 3.971 | .000 |
|  | > 80 | 2.183 | 1.826 | 2.610 | .000 |
| Race | American | 1.000 | 1.000 | 1.000 |  |
|  | Asian or | .720 | .413 | 1.256 | .247 |
|  | Black | .949 | .813 | 1.108 | .507 |
|  | Unknown | .792 | .661 | .949 | .011 |
|  | White | .632 | .369 | 1.084 | .096 |
| Primary_site | Proximal | 1.000 | 1.000 | 1.000 |  |
|  | Distal | 1.893 | 1.674 | 2.140 | .000 |
| Grade | 1 | 1.000 | 1.000 | 1.000 |  |
|  | 2 | .502 | .299 | .844 | .009 |
|  | 3 | .728 | .601 | .883 | .001 |
|  | 4 | 1.087 | .946 | 1.249 | .240 |
|  | Unknown | 1.070 | .551 | 2.075 | .842 |
| Lauren classification | Intestinal | 1.000 | 1.000 | 1.000 |  |
|  | Diffused | .795 | .623 | 1.016 | .067 |
|  | Unspecified | 1.601 | 1.237 | 2.072 | .000 |
| Chemotherapy | Yes | 1.000 | 1.000 | 1.000 |  |
|  | No/Unkown | .662 | .585 | .748 | .000 |
| Surgery | Yes | 1.000 | 1.000 | 1.000 |  |
|  | No/Unkown | 11.041 | 9.379 | 12.997 | .000 |
| Radiation | Yes | 1.000 | 1.000 | 1.000 |  |
|  | No/Unkown | 2.635 | 2.245 | 3.092 | .000 |

| **Table S12. Logistic analysis of peritoneal metastasis in Nanfang Hospital cohort** | | | | | |
| --- | --- | --- | --- | --- | --- |
| **Variable** | **Subgroup** | **OR** | **Lower** | **Upper** | ***P*** |
| Sex | Female | 1.000 | 1.000 | 1.000 |  |
|  | Male | .839 | .640 | 1.101 | .206 |
| Age | 18 – 39 | 1.000 | 1.000 | 1.000 |  |
|  | 40 – 59 | 1.479 | .604 | 3.618 | .392 |
|  | 60 – 79 | 1.277 | .551 | 2.957 | .568 |
|  | > 80 | .890 | .388 | 2.046 | .784 |
| Primary_site | Proximal | 1.000 | 1.000 | 1.000 |  |
|  | Distal | 1.789 | 1.264 | 2.534 | .001 |
| Grade | 1 | 1.000 | 1.000 | 1.000 |  |
|  | 2 | .239 | .086 | .666 | .006 |
|  | 3 | .413 | .213 | .802 | .009 |
|  | 4 | .735 | .418 | 1.292 | .285 |
|  | Unknown | .0000 | .0000 |  | 0.999 |
| Lauren classification | Intestinal | 1.000 | 1.000 | 1.000 |  |
|  | Diffused | 1.080 | .722 | 1.614 | .708 |
|  | Unspecified | 1.272 | .921 | 1.757 | .144 |
| Chemotherapy | Yes | 1.352 | .767 | 2.383 |  |
|  | No/Unkown | .371 | .278 | .497 | .000 |
| Surgery | Yes | 1.0000 | 1.0000 | 1.0000 |  |
|  | No/Unkown | 7.235 | 5.481 | 9.550 | .000 |
| Radiation | Yes | 1.0000 | 1.0000 | 1.0000 |  |
|  | No/Unkown | 1.550 | .719 | 3.339 | .263 |

| **Table S13. Logistic analysis of ovary metastasis in Nanfang Hospital cohort** | | | | | |
| --- | --- | --- | --- | --- | --- |
| **Variable** | **Subgroup** | **OR** | **Lower** | **Upper** | ***P*** |
| Sex | Female | 1.000 | 1.000 | 1.000 |  |
|  | Male | 0 | 0 |  | .987 |
| Age | 18 – 39 | 1.000 | 1.000 | 1.000 |  |
|  | 40 – 59 | 0 | 0 |  | .997 |
|  | 60 – 79 | 0 | 0 |  | .997 |
|  | > 80 | 0 | 0 |  | .998 |
| Primary_site | Proximal | 1.000 | 1.000 | 1.000 |  |
|  | Distal | 1.308 | .356 | 4.800 | .686 |
| Grade | 1 | 1.000 | 1.000 | 1.000 |  |
|  | 2 | 0 | 0 |  | .996 |
|  | 3 | 1.617 | 0 |  | 1.000 |
|  | 4 | 0 | 0 |  | .997 |
|  | Unknown | .0000 | .0000 |  | 0.999 |
| Lauren classification | Intestinal | 1.000 | 1.000 | 1.000 |  |
|  | Diffused | .869 | .296 | 2.555 | .799 |
|  | Unspecified | .383 | .164 | .897 | .027 |
| Chemotherapy | Yes | 1.352 | .767 | 2.383 |  |
|  | No/Unkown | .445 | .184 | 1.073 | .071 |
| Surgery | Yes | 1.0000 | 1.0000 | 1.0000 |  |
|  | No/Unkown | 2.734 | 1.269 | 5.889 | .010 |
| Radiation | Yes | 1.0000 | 1.0000 | 1.0000 |  |
|  | No/Unkown | .927 | .095 | 9.034 | .948 |

| **Table S14. Logistic analysis of pleura metastasis in Nanfang Hospital cohort** | | | | | |
| --- | --- | --- | --- | --- | --- |
| **Variable** | **Subgroup** | **OR** | **Lower** | **Upper** | ***P*** |
| Sex | Female | 1.000 | 1.000 | 1.000 |  |
|  | Male | .538 | .140 | 2.064 | .366 |
| Age | 18 – 39 | 1.000 | 1.000 | 1.000 |  |
|  | 40 – 59 | .000 | .000 | . | .995 |
|  | 60 – 79 | .076 | .010 | .582 | .013 |
|  | > 80 | .081 | .012 | .561 | .011 |
| Primary_site | Proximal | 1.000 | 1.000 | 1.000 |  |
|  | Distal | 1.182 | .246 | 5.670 | .835 |
| Grade | 1 | 1.000 | 1.000 | 1.000 |  |
|  | 2 | .000 | .000 | . | .997 |
|  | 3 | 1.592 | .133 | 19.057 | .713 |
|  | 4 | .659 | .069 | 6.323 | .718 |
|  | Unknown | .000 | .000 | . | .999 |
| Lauren classification | Intestinal | 1.000 | 1.000 | 1.000 |  |
|  | Diffused | .730 | .116 | 4.583 | .737 |
|  | Unspecified | .652 | .135 | 3.153 | .595 |
| Chemotherapy | Yes | 1.352 | .767 | 2.383 |  |
|  | No/Unkown | .257 | .045 | 1.472 | .127 |
| Surgery | Yes | 1.0000 | 1.0000 | 1.0000 |  |
|  | No/Unkown | 3.998 | .921 | 17.360 | .064 |
| Radiation | Yes | 1.0000 | 1.0000 | 1.0000 |  |
|  | No/Unkown | .351 | .038 | 3.223 | .355 |


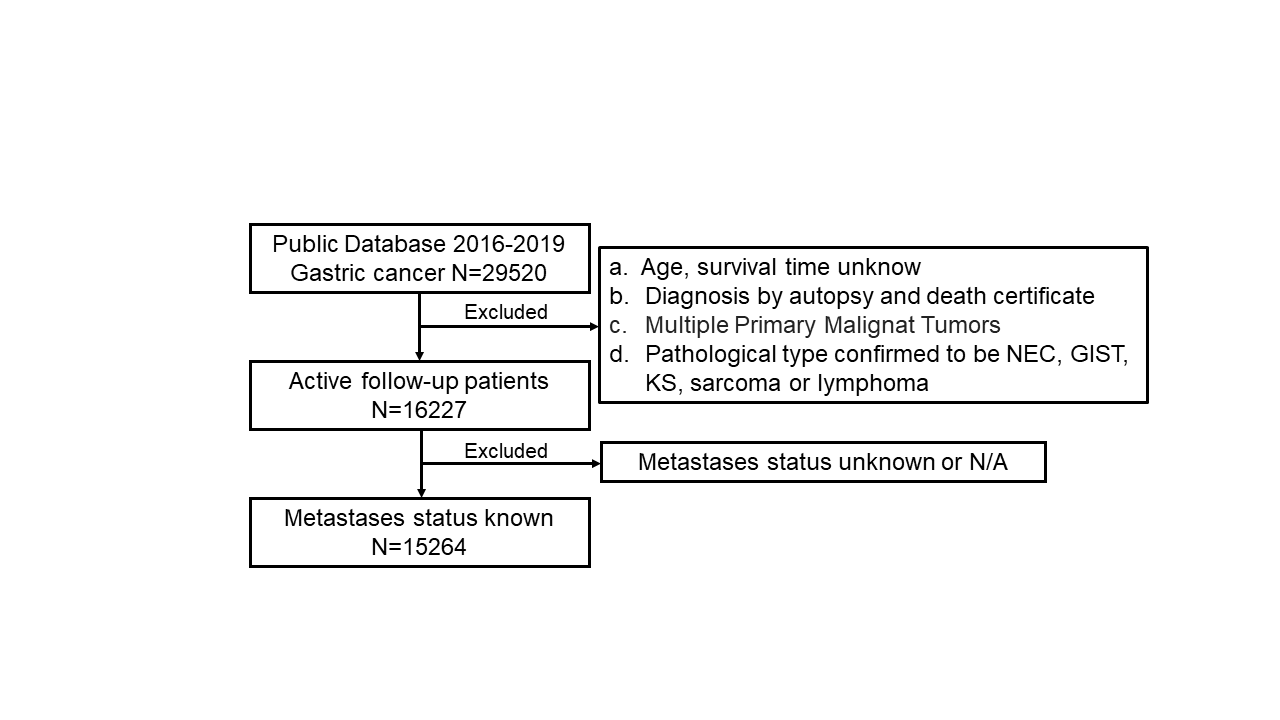


**Figure S1. Flowchart of the patient selection process in SEER cohort of this study.**


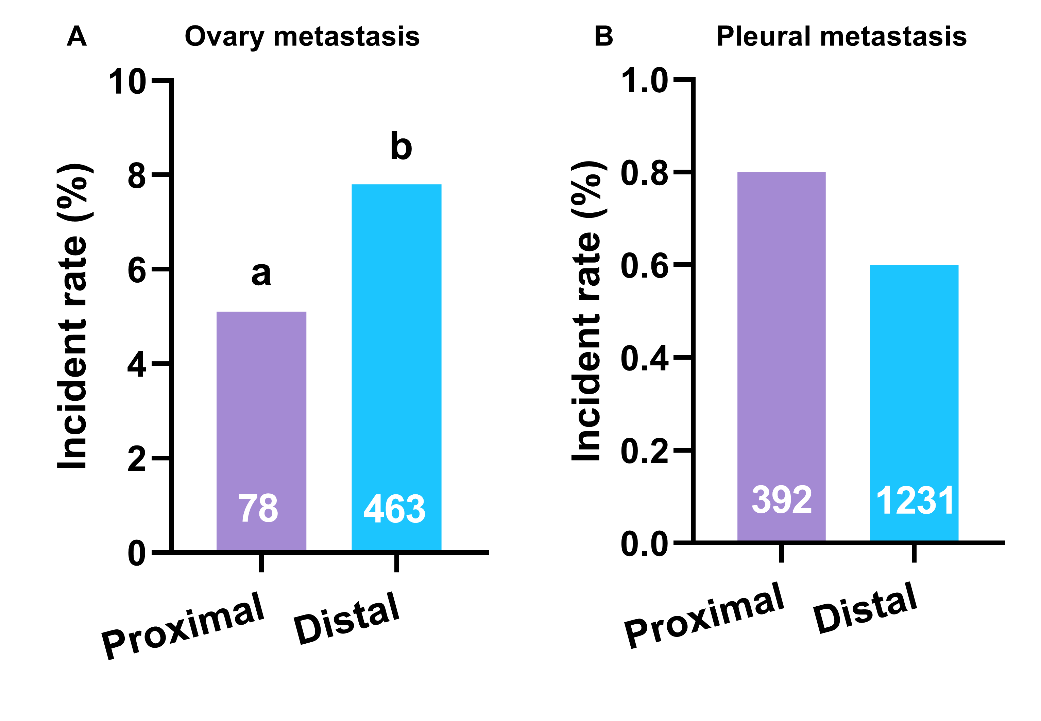


**Figure S2. The metastatic incidences of (A) ovary and (B) pleural between proximal and distal gastric cancers in the Nanfang Hospital cohort.**
